# Natural Killer-Like B Cells Secreting Interleukin-18 Induces a Proinflammatory Response in Periodontitis

**DOI:** 10.3389/fimmu.2021.641562

**Published:** 2021-02-18

**Authors:** Ye Zhang, Wei Kuang, Danfeng Li, Yu Li, Yi Feng, Xinwei Lyu, Gao-Bo Huang, Jian-Qi Lian, Xiao-Fei Yang, Cheng Hu, Yajuan Xie, Song Xue, Jiali Tan

**Affiliations:** ^1^Department of Orthodontics, Hospital of Stomatology, Sun Yat-sen University, Guangzhou, China; ^2^Guangdong Provincial Key Laboratory of Stomatology, Sun Yat-sen University, Guangzhou, China; ^3^Guanghua School of Stomatology, Sun Yat-sen University, Guangzhou, China; ^4^Department of Infectious Diseases, Tangdu Hospital, Fourth Military Medical University, Xi'an, China; ^5^Guangzhou Key Laboratory of Basic and Applied Research of Oral Regenerative Medicine, Department of Oral and Maxillofacial Surgery, Affiliated Stomatology Hospital of Guangzhou Medical University, Guangzhou, China; ^6^Department of Infectious Diseases, Shaanxi Provincial People's Hospital, The Affiliated Hospital of Xi'an Medical University, Xi'an, China; ^7^Department of Hepatobiliary Surgery, Institute of Advanced Surgical Technology and Engineering, The First Affiliated Hospital of Xi'an Jiaotong University, Xi'an, China; ^8^Department of Anesthesiology, Xijing Hospital, Fourth Military Medical University, Xi'an, China

**Keywords:** periodontitis, *Porphyromonas gingivalis*, animal model, natural killer-like B cells, interleukin-18

## Abstract

Natural killer-like B (NKB) cells, which are newly identified immune subsets, reveal a critical immunoregulatory property in the eradication of microbial infection *via* the secretion of interleukin (IL)-18. For the first time, this study investigated the role of NKB cells in secreting IL-18 in the pathogenesis of periodontitis. In this study, NKB cells' percentage and IL-18 concentration in peripheral blood and periodontium in periodontitis patients was measured using flow cytometry and ELISA. The role of IL-18 in regulating periodontal inflammation was examined in a *Porphyromonas gingivalis* (*P. gingivalis*)-induced periodontitis murine model. Peripheral and periodontal-infiltrating CD3^−^CD19^+^NKp46^+^ NKB cells, which were the main source of IL-18, were elevated and correlated with attachment loss in periodontitis patients. *In vitro* IL-18 stimulation promoted proinflammatory cytokine production in periodontal ligament cells. *P. gingivalis* infection induced elevation of IL-18 receptor in periodontium in a periodontitis murine model. IL-18 neutralization not only suppressed *P. gingivalis*-induced alveolar bone resorption, but also inhibited recruitment of antigen-non-specific inflammatory cells into the periodontium, probably *via* dampening expressions of cytokines, chemokines, and matrix metalloproteinases. NKB cells secreting IL-18 appeared to be an important mediator in the inflammatory response following intraoral *P. gingivalis* infection. These findings might be relevant to the development of immunotherapies for periodontitis.

## Introduction

Periodontitis is an intraoral infection-driven inflammatory disease in periodontal supporting tissues that leads to the pathologic loss of the periodontal ligament and alveolar bone, and even loosening of the teeth (1, 2). *Porphyromonas gingivalis* (*P. gingivalis*) is one of the major periodontopathic pathogens. Host inflammatory and immune responses to microbial communities alter the subgingival microenvironment, inducing *P. gingivalis* to be the dominant bacteria in the biofilm. This process breaks the homeostasis between symbiotic microorganisms and the immune system, promotes the development of periodontitis, and triggers systemic diseases (3). Immune cell activation and cytokines/chemokines secretion played important roles in defining the stabilization or progression of lesions during periodontal inflammation (4).

Natural killer-like B (NKB) cells, which differentiate from bone marrow pro-B cells, are a newly identified lymphocyte subset distinct from NK cells and B cells (5). NKB cells appear in spleen and mesenteric lymph nodes of mice (phenotype: CD3^−^NK1.1^+^CD19^+^) and humans (phenotype: CD3^−^NKp46^+^CD19^+^) (5), and function as a separate subset of innate B cells (6). NKB cells expanded rapidly within 24 h and activate innate lymphocytes to protect against microbial infection *via* secretion of interleukin (IL)-18 and IL-12 in response to bacterial infection (5). IL-18 was accumulated in gingival tissues, gingival crevicular fluids (GCFs), and serum of patients with chronic and aggressive periodontitis (7–10). Recombinant human IL-18 stimulation promoted the secretion of matrix metalloproteinases (MMPs) in human periodontal ligament fibroblasts *in vitro*, which played a pivotal role in the development of periodontitis (11). Furthermore, IL-18 transgenic mouse presented increased periodontal bone loss in *P. gingivalis*-induced periodontitis *in vivo* (12), indicating divergent activity of NKB cells secreting IL-18 in response to bacterial infection. Monocytes/macrophage and NKB cells are a source of IL-18; IL-18 can induce proinflammatory cytokine production and gene expression, yet is mainly protective in microbial infections. We thus hypothesized that NKB cells producing IL-18 may also perform a pivotal role in the immunopathogenesis of periodontitis. To test this possibility, we examined NKB cells and secreting cytokines in periodontitis patients, and also assessed the ability of IL-18 to evoke periodontal inflammation in *P. gingivalis*-induced periodontitis mice.

## Materials and Methods

### Subjects

The current study protocol involving human participants was approved by the Ethics Committee of Hospital of Stomatology, Sun Yat-sen University (Guangzhou, Guangdong Province, China) [Approval No. ERC-(2016)-39]. Written informed consent was obtained from each enrolled subject. Clinical parameters included clinical attachment level (CAL), probing depth (PD), and bleeding on probing (BOP). The diagnosis of periodontitis was made according to the new classification and case definition revised in 2018 (13). A patient was defined as a periodontitis case in the context of clinical care if interdental CAL was detectable in no less than two non-adjacent teeth, or when buccal or palatal CAL no less than 3 mm with PD more than 3 mm was detectable in no less than two teeth (13). All diagnosed periodontitis patients were followed-up every 2–3 months. During the follow-up period, patients with CAL exacerbation more than 2 mm who tested strong positive for BOP were defined as acute phase. Patients with CAL exacerbation less than 2 mm and were negative/weak positive for BOP were defined as maintenance phase. A total of 38 patients with periodontitis, including 12 patients in acute phase and 26 patients in maintenance phase, were enrolled. The clinical data of enrolled patients are shown in Table S1. Blood sampling was made when the patients were defined as in the acute or maintenance phase. Ten milliliters of ethylene diamine tetraacetic acid anti-coagulated whole blood was collected from the median cubital vein. Peripheral blood mononuclear cells (PBMCs) were then isolated by Ficoll-Hypaque (Sigma-Aldrich, St. Louis, MO, USA) density gradient centrifugation. Before GFCs sampling, supragingival plaque was gently removed, and the tooth surface was air-dried. A dental absorbent paper point was gently inserted and held in the gingival sulcus at the periodontitis site for 30 s. This process was repeated twice for each sampling site. The absorbent paper points were dipped in 100 μL of PBS for 1 h. GFCs were stored at −80°C until use (14). Biopsy of periodontium was harvested from a periodontitis site using a 3 mm diameter punch. Following sampling, patients underwent conventional periodontal therapy consisting of full mouth scaling and root planning with ultrasonic and manual instruments. 0.2% of chlorhexidine mouthwash was prescribed twice daily for 30 days. Peripheral blood and GFCs samples were performed in all patients in the maintenance phase post therapy. Fifteen sex- and age-matched healthy individuals, who underwent routine premolar procedures for orthodontic reasons or third molar extraction, were also enrolled as controls. Periodontal ligament tissues were obtained from healthy controls following premolar extraction. All subjects have a minimum of twenty natural teeth (excluding third molars). No subjects were afflicted with systemic diseases (including chronic viral infection, autoimmune disease, or malignancy), were pregnant, or were taking hormonal contraceptives. Individuals who received orthodontic treatment or periodontal therapy before the study, or who received systemic antibiotics therapy within the past 3 months, were excluded from the study.

### Induction of Periodontitis in Mice

All animal experiment procedures were conducted according to the protocol approved by the Institutional Animal Care and Use Committee of Sun Yat-sen University (Guangzhou, Guangdong Province, China) (Approval No. SYSU-IACUC-2019-000320). Six- to eight-week-old wild type male C57BL/6 mice were used in this study. Mice were given sulfamethoxazole/trimethoprim *ad libitum* in the drinking water for 10 days, followed by a 3-day antibiotic-free period. Mice were then infected with 10^9^ colony-forming units of live *P. gingivalis* strain ATCC 33277 (15, 16) in 100 μL PBS with 2% carboxymethylcellulose by gavage three times at 2–4-day intervals. Furthermore, a ligature of 6–0 silk suture saturated with *P. gingivalis* was also slightly applied to the cervical region of the maxillary second molar teeth on both sides, which only confirmed the *P. gingivalis* adhesion to periodontium but did not induce significant mechanical trauma. The silk suture was displaced apically into the gingival sulcus weekly to ensure it maintained a subgingival position, and was replaced as necessary (17–19). For the acute phase of the periodontitis model, mice also received local palatal gingival microinjection with 50 μL of lipopolysaccharide (LPS; 1 mg/mL; InvivoGen, San Diego, CA) every other day, and were sacrificed by excessive anesthesia 14 days after first gavage (20, 21). For the maintenance phase of the periodontitis model, mice were maintained for 4 weeks by gavage and ligature, and were sacrificed by excessive anesthesia 42 days after first gavage. Uninfected control mice received carboxymethylcellulose in PBS. Infected mice were kept in a separate quarantine room away from the uninfected control mice, but under the same 12-h/12-h light/dark cycle and a constant temperature of 25°C. In certain experiments, mice were also microinjected locally by anti-mouse IL-18 antibody (R&D Systems, Minneapolis, MN). Microinjection of anti-IL-18 was performed on the mesial of the first molar and in the papillae between the first and second and third molars on both sides of the maxilla using a microfine needle (BD Biosciences, San Jose, CA) on days 4, 9, 11, 13, 20, 27, and 34 post initial infection. Anti-mouse IL-18 antibody was diluted in PBS for a final concentration of 5 μg/mL. The volume for each injection was 15 μL for each site. Each treatment group contained four or six animals.

### Isolation of Periodontal Ligament Cells and Periodontal-Infiltrating Leukocytes

For periodontal ligament cells' isolation, tissue was obtained from the periodontal ligament of the root surface and was digested by collagenase I (0.66 mg/mL) at 37°C for more than 20 min for single-cell suspension. Periodontal ligament cells, which were positive for vimentin and negative to keratin, were harvested at passage 3, and were stimulated with recombinant human IL-18 (1 μg/mL; Abcam, Cambridge, MA) for 48 h. For periodontal-infiltrating leukocytes isolation, periodontium were digested by collagenase I (0.66 mg/mL) at 37°C for 1 h and passed through a 40 μm strainer. ACK lysis buffer was added for red blood cells lysis. Periodontal-infiltrating leukocytes were then obtained by centrifugation at 3000 × *g* for 10 min at 4°C.

### Flow Cytometry

PBMCs and periodontal-infiltrating leukocytes from enrolled subjects were surface-stained with anti-human CD3-PerCP (BD Biosciences, San Jose, CA), CD19-APC-H7 (BD Pharmingen), NKp46-PE-Cy7 (BD Pharmingen), and CD14-FITC (eBioscience, San Diego, CA), and were intracellularly stained with anti-human IL-18 (Abcam), which was conjugated by PE/R-Phycoerythrin Conjugation Kit (Abcam). PBMCs, splenocytes, and periodontal-infiltrating leukocytes from mice were surface-stained with anti-mouse CD3-FITC, CD4-APC, CD8-PerCP Cy5.5, NK1.1-PE, CD11b-FITC, CD11c-PE, CD19-APC, and/or Ly-6G (Gr-1)-PE Cy7 (eBioscience) for detection of leukocyte subsets. Periodontal ligament cells were stained with Annexin V-FITC and propidium iodide. Data were acquired using FACS Aria II flow cytometer (BD Biosciences) and were analyzed using FlowJo Version 10 (Tree Star, Ashland, OR).

### Cellular Proliferation Assay

Cellular proliferation was measured by Cell Counting Kit-8 (CCK-8; Beyotime Biotechnology, Wuhan, Hubei Province, China).

### Enzyme-Linked Immunosorbent Assay (ELISA)

IL-18 and IL-12 levels in the serum and GCF were measured by Human Total IL-18 Quantikine QuicKit ELISA (R&D Systems) and Human IL-12 p70 Quantikine ELISA kit (R&D Systems), respectively. Interferon-γ (IFN-γ), granulocyte macrophage-colony stimulating factor (GM-CSF), IL-2, and tumor necrosis factor-α (TNF-α) levels in the cultured supernatants were measured by Human IFN-γ Quantikine ELISA kit (R&D Systems), Human GM-CSF Quantikine ELISA kit (R&D Systems), Human IL-2 Quantikine ELISA kit (R&D Systems), and Human TNF-α Quantikine ELISA kit (R&D Systems), respectively.

### Micro-CT Assay

Formalin-fixed maxillae were subjected to micro-CT image analysis. The specimens were scanned by Scanco μCT50 scanner (Scanco Medical AG, Brutishly, Switzerland) using a voxel size of 18 μm at 70k Vp and 200 μA. The Materialize Mimics v17.0 Software was used for reconstruction, visualization, and quantification. The length between the cementoenamel junction and alveolar bone crest (CEJ-ABC) at two sites for the first molars (distopalatal and distobuccal) and two sites for the second molars (mesiopalatal and mesiobuccal) in three-dimensional images was measured to assess alveolar bone loss (22). Cortical bone mineral density (BMD) was also assessed.

### Histopathologic Examination

Histological analysis was performed as previously described (17). The degree of inflammation was determined by counting leukocytes at the site with the most intense inflammation and calculated as number of leukocytes/10,000 μm^2^ within the gingival epithelium and adjacent subepithelial layer.

### Real-Time Reverse-Transcription PCR

Total periodontium RNA was extracted by Trizol (Invitrogen, ThermoFisher, Carlsbad, CA, USA). RNA was reversely transcribed with random prime by M-MLV Reverse Transcriptase (Promega, Madison, WI, USA). Real-time polymerase chain reaction was performed using an Applied Biosystems 7,500 Real-Time PCR System by Platinum SYBR Green qPCR SuperMix (Invitrogen). Relative gene expression was semi-quantified by the Δ*ΔC*_*T*_ method using 7,500 Sequence Detection Software (Applied Biosystems, Foster City, CA, USA).

### Statistical Analysis

Data were analyzed using SPSS Version 21.0 (SPSS, Chicago, IL, USA). Statistical significance was determined by Student *t*-test, One-Way ANOVA, SNK-*q* test, Dunn's multiple comparison test, paired *t*-test, or Wilcoxon paired test. *P* < 0.05 were considered as significant differences.

## Results

### Elevated NKB Cells and IL-18 Production in Periodontitis Patients

**Figure 1:**
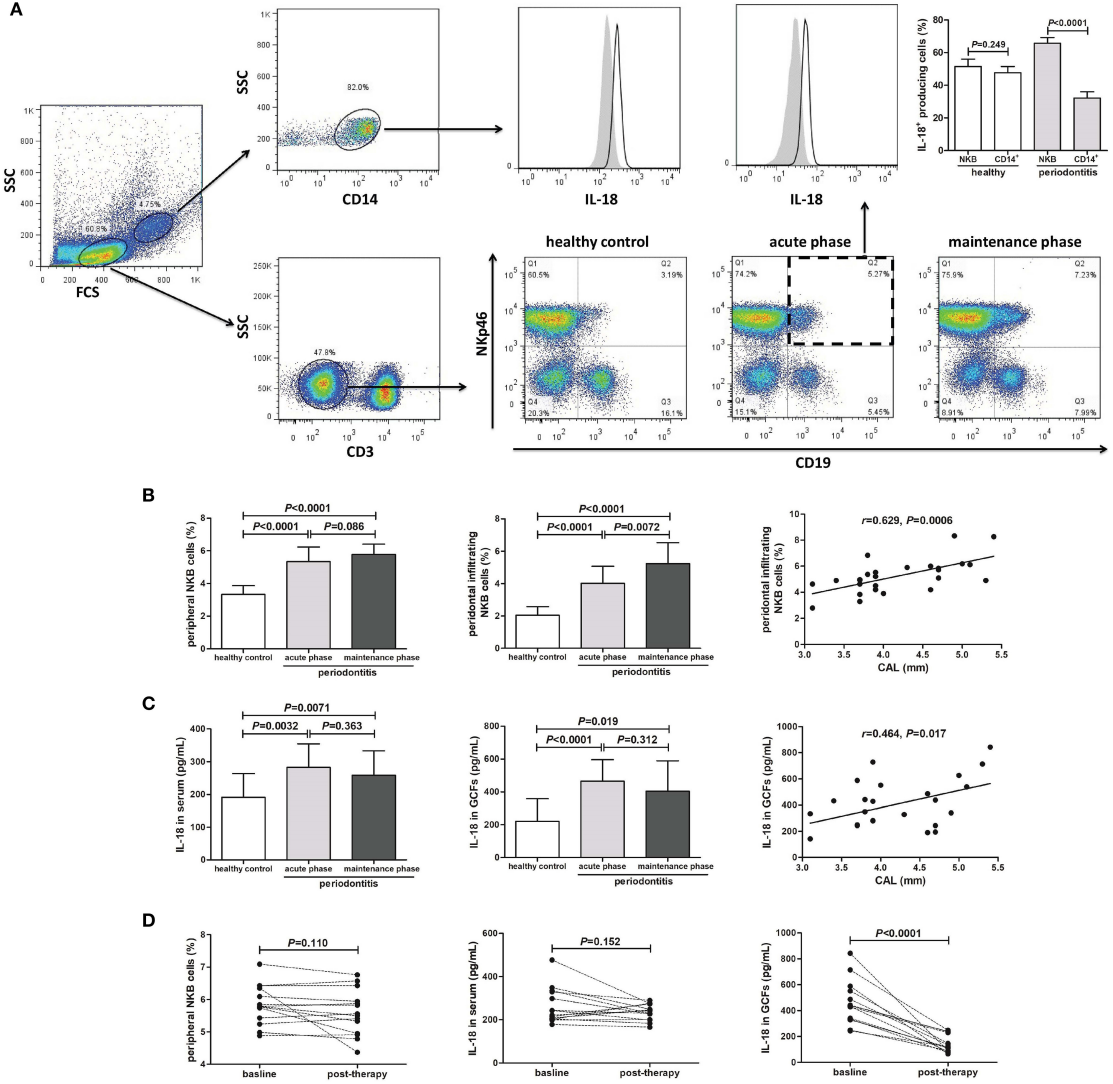
Peripheral and periodontal-infiltrating natural killer-like B (NKB) cells secreting IL-18 in human periodontitis. **(A)** Representative FACS plots of CD3^−^CD19^+^NKp46^+^ NKB cells in healthy volunteers (*n* = 15), acute phase of periodontitis patients (*n* = 12), and maintenance phase of periodontitis patients (*n* = 26). IL-18 production by CD14^+^ monocytes and NKB cells was shown. IL-18^+^ cellular sources in the periodontium of healthy volunteers and periodontitis patients was compared (mean ± standard deviation, Student *t*-test). **(B)** Peripheral and periodontal infiltrating NKB cells' percentage among groups (SNK-*q* test), and correlation between periodontal infiltrating NKB cells and attachment loss (AL) in maintenance phase of periodontitis patients (Pearson correlation analysis). **(C)** IL-18 concentration in serum and GCFs among groups (Dunn's multiple comparison test), and correlation between IL-18 level in GCFs and AL in maintenance phase of periodontitis patients (Spearman correlation analysis). **(D)** Peripheral NKB cells, serum, and GCFs IL-18 concentration in response to periodontal treatment in periodontitis patients (*n* = 14) (paired *t*-test or Wilcoxon paired test).

We examined the peripheral blood and periodontal infiltrating NKB cells and secreting cytokine in 12 patients with an acute phase of periodontitis, 26 patients with a maintenance phase of periodontitis, and 15 healthy individuals. PBMCs and periodontal-infiltrating leukocytes were separated using the gates shown by flow cytometry. The larger gated population was made up of monocytes, while the smaller cells were lymphocytes. The CD19^+^NKp46^+^ cells within CD3^−^ lymphocytes were defined as NKB cells (5) (Figure 1A). Both NKB cells and CD14^+^ monocytes were the main source of IL-18 (Figure 1A). There was no significant difference of IL-18^+^-producing cells between NKB cells and CD14^+^ monocytes in periodontal-infiltrating leukocytes from healthy volunteers. However, NKB cells presented the major source of IL-18 in the periodontium of periodontitis patients (Figure 1A). The percentage of both peripheral and periodontal infiltrating CD3^−^CD19^+^NKp46^+^ NKB cells was robustly elevated in patients with periodontitis (Figure 1B). There was no significant difference of peripheral NKB cells between acute and maintenance phases. Periodontal-infiltrating NKB cells were increased in the maintenance phase of periodontitis, and positively correlated with CAL (Figure 1B). There were no statistical differences of either peripheral or periodontal IL-12 expression between healthy controls and periodontitis patients (Supplementary Figure 1). Both IL-18 level in the serum and GCFs were elevated in periodontitis patients, but were comparable between acute and maintenance phases (Figure 1C). There was a positive correlation between IL-18 concentration in GCFs and CAL in the maintenance phase of periodontitis (Figure 1C). Peripheral NKB cells and IL-18 did not change significantly in response to therapy (Figure 1D). However, IL-18 expression in GCFs was notably down-regulated post-treatment (Figure 1D).

### IL-18 Promoted Inflammatory Reaction of Periodontal Ligament Cells

**Figure 2:**
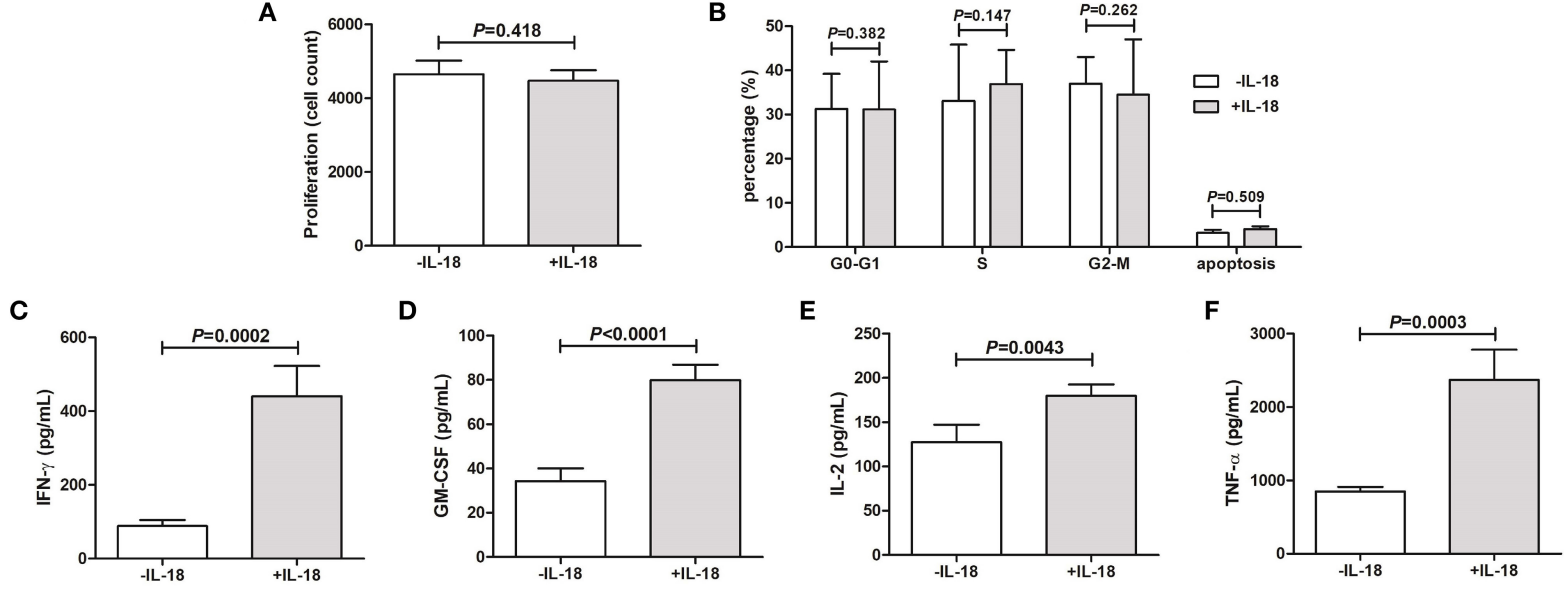
IL-18 regulates the inflammatory reaction of periodontal ligament cells *in vitro*. Periodontal ligament cells were isolated from five healthy individuals who underwent routine premolar procedures for orthodontic reasons and were stimulated with recombinant human IL-18 for 48 h. **(A)** Proliferation of periodontal ligament cells was measured by Cell Counting Kit-8. **(B)** Cell cycle and apoptosis was assessed by flow cytometry. Expression of **(C)** Interferon-γ (IFN-γ), **(D)** granulocyte macrophage-colony stimulating factor (GM-CSF), **(E)** IL-2, and **(F)** tumor necrosis factor-α (TNF-α) was measured by ELISA. Data are expressed as mean ± standard deviation. Group differences were assessed by paired *t*-test.

Periodontal ligament cells were isolated from five healthy individuals who underwent routine premolar procedures for orthodontic reasons. Purified periodontal ligament cells were stimulated with or without IL-18 stimulation *in vitro* for 48 h. The growth of IL-18-stimulated periodontal ligament cells was comparable with unstimulated cells (Figure 2A). Flow cytometry was also performed to assess the percentage of periodontal ligament cells in different cell cycles and apoptosis. There were no significant differences in the percentage of cells in G0-G1 phase, S phase, G2-M phase, or apoptosis (Figure 2B). The concentration of proinflammatory cytokines, including IFN-γ, GM-CSF, IL-2, and TNF-α, was assessed by ELISA. All above cytokines were robustly elevated in response to IL-18 stimulation (Figures 2C–F).

### IL-18 Neutralization Ameliorated Alveolar Bone Loss and Inflammatory Reaction

**Figure 3:**
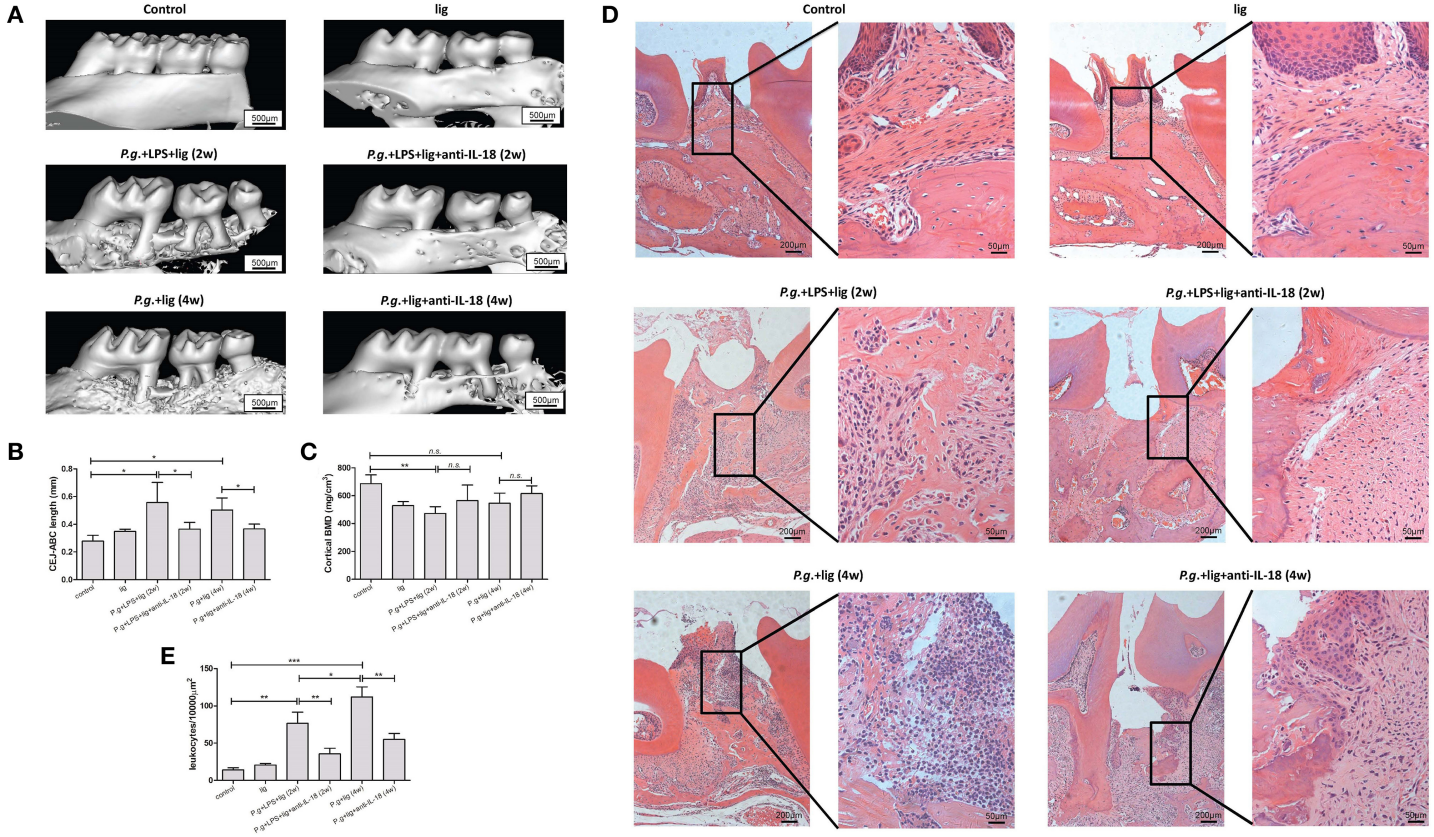
IL-18 neutralization ameliorates alveolar bone loss and reduced inflammation in periodontitis murine models. **(A)** Representative micro-CT three-dimensional images viewed from the buccal side of maxillae in untreated control mice (*upper left panel, n* = 3), silk ligature mice (*upper right panel, n* = 3), mice given LPS injection and *P. gingivalis* infection for 2 weeks (*middle left panel, n* = 5), mice given *P. gingivalis* infection for 4 weeks (*lower left panel, n* = 4), and mice with anti-IL-18 antibody administration (*lower left panel, n* = 3; *lower right panel, n* = 3). **(B)** Quantification of cementoenamel junction and alveolar bone crest (CEJ-ABC) length which indicated alveolar bone loss, and **(C)** cortical bone mineral density (BMD) was measured using reconstructed three-dimensional images. **(D)** Representative histological sections of periodontium of control and periodontitis mice (hematoxylin and eosin staining; magnification, × 100 and × 400, respectively). **(E)** Quantification of infiltrating inflammatory leukocytes in periodontium. Data are expressed as mean ± standard deviation. Group differences were assessed by SNK-*q* tests. *P < 0.05, **P < 0.01, ***P < 0.001, *n.s*., no significance; lig, ligation; *P.g., P. gingivalis*.

*P. gingivalis* infection and/or LPS injection induced the ~2-fold induction of IL-18Rα and IL-18Rβ mRNA in periodontium (Supplementary Figure 2), indicating increased sensitivity to IL-18 in periodontitis. Silk ligature alone did not induce notable alveolar bone resorption (Figures 3A,B). Following injection with LPS and infection with *P. gingivalis*, bone loss was robustly increased by ~50% (Figures 3A,B, Supplementary Figure 3). CEJ-ABC length was comparable between the combination of 2-week LPS injection + *P. gingivalis* infection mice and 4-week *P. gingivalis* infection mice (Figure 3B). Administration of anti-IL-18 antibody reduced bone resorption in both periodontitis murine models by ~30−40% (Figures 3A,B, Supplementary Figure 3). With regard to cortical BMD, LPS infection in combination with *P. gingivalis* infection induced down-regulation of BMD (Figures 3A,C, Supplementary Figure 3). In contrast, *P. gingivalis* infection alone did not decrease BMD when compared with control (Figures 3A,C, Supplementary Figure 3). IL-18 neutralization slightly up-regulated BMD in both models, however, these differences failed to achieve statistical significances (*P* = 0.104 and = 0.253, respectively, Figure 3C).

Consistent with the micro-CT assay, in control mice without treatment or in mice with silk ligature, there was no evidence of a significant inflammatory reaction or other pathological alterations in the periodontium (Figure 3D, *upper panel*). Histological examination revealed inflammatory leukocytes' recruitment in the connective tissue and surrounding bone tissue with associated proliferation of junctional epithelium. Importantly, these periodontium presented more inflammatory cells infiltration in LPS + *P. gingivalis* treated mice (Figure 3D, *middle left panel*) than *P. gingivalis* infected mice (Figure 3D, *lower left panel*) (Figure 3E). Administration of anti-IL-18 antibody reduced inflammatory reactions in both models (Figure 3D, *middle and lower right panel*). A quantitative measure of periodontal histopathology was markedly decreased by anti-IL-18 antibody administration after LPS injection and *P. gingivalis* infection (Figure 3E).

### IL-18 Neutralization Blocked Inflammatory Cells Recruitment and Reduced Cytokine/Chemokine Expression in Periodontium

**Figure 4:**
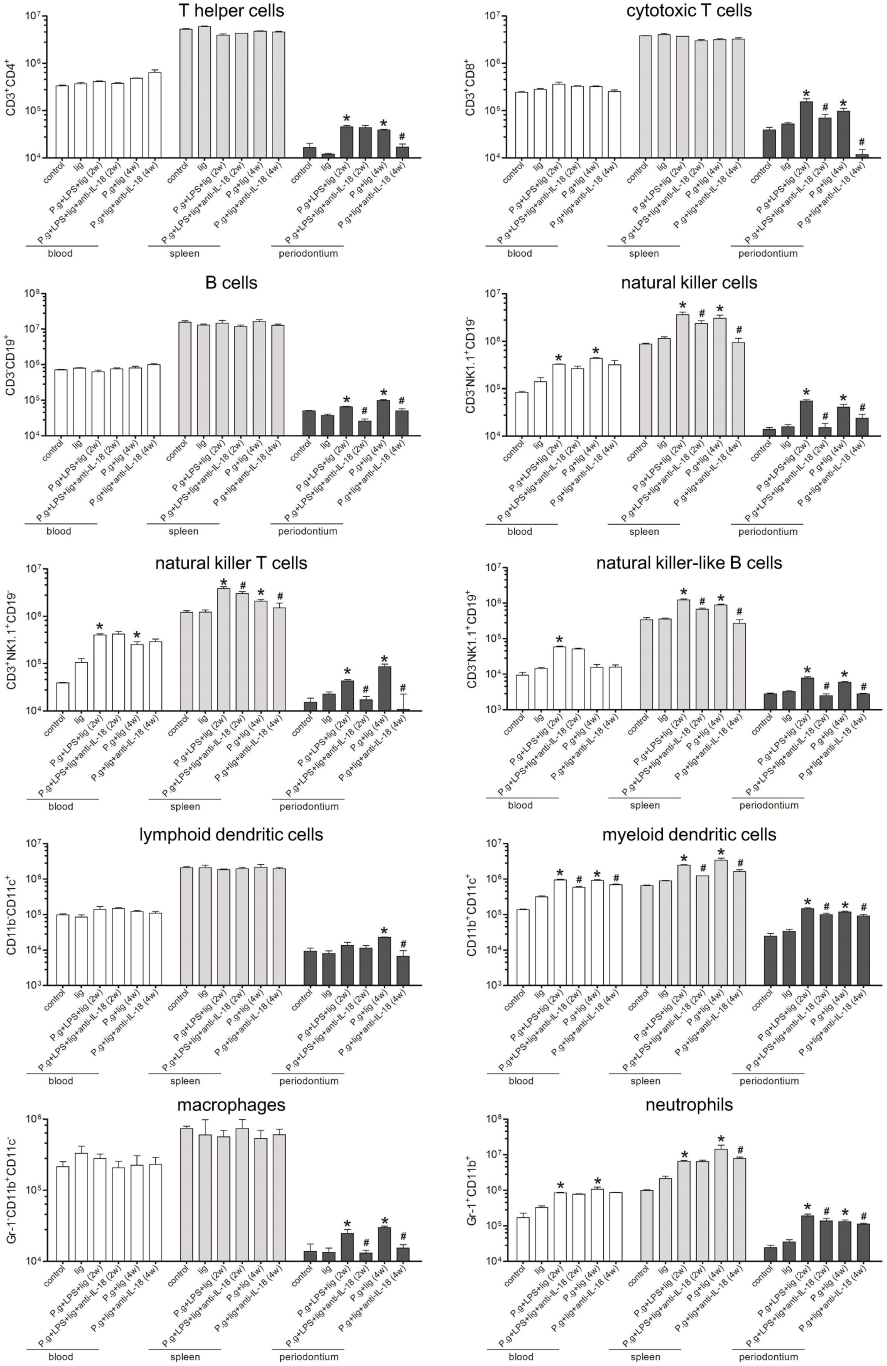
Depletion of IL-18 blocks the infiltration of antigen-non-specific cells into the periodontium. Periodontium from untreated control mice (*n* = 3), silk ligature mice (*n* = 3), mice given LPS injection and *P. gingivalis* infection for 2 weeks (*n* = 6), mice with anti-IL-18 antibody administration (*n* = 4), mice given *P. gingivalis* infection for 4 weeks (*n* = 5), and mice with anti-IL-18 antibody administration (*n* = 4) were weighed at the time of sacrifice, and total leukocytes were isolated from periodontium of similar weights and analyzed by flow cytometry. The indicated numbers represent the absolute numbers of cell subsets in the peripheral blood, spleen, and periodontium, respectively. Data are expressed as mean ± standard deviation. Group differences were assessed by SNK-*q* tests. *P < 0.05 compared with controls. ^#^*P* < 0.05 compared with mice without anti-IL-18 antibody administration. lig, ligation; *P.g., P. gingivalis*.

To determine the cellular makeup of the inflammatory recruitment in the periodontium, the absolute numbers of peripheral blood, splenocytes, and periodontal-infiltrating cells were quantified. Silk ligature did not induce inflammatory cell subsets in peripheral blood, spleens, or periodontium. When compared with controls, there were no remarkable differences in T helper cells (Th cells; CD3^+^CD4^+^), cytotoxic T cells (CD3^+^CD8^+^), B cells (CD3^−^CD19^+^), lymphoid dendritic cells (CD11b^−^CD11c^+^), or macrophages (CD11b^+^CD11c^−^Gr-1^−^) in the peripheral blood or spleens of *P. gingivalis* infected mice with or without anti-IL-18 antibody. LPS injection and *P. gingivalis* infection increased natural killer cells (NK cells, CD3^−^NK1.1^+^CD19^−^), NKT cells (CD3^+^NK1.1^+^CD19^−^), NKB cells (CD3^−^NK1.1^+^CD19^+^), myeloid dendritic cells (CD11b^+^CD11c^+^), and neutrophils (CD11b^+^Gr-1^+^) in both peripheral blood and spleens, however, administration of anti-IL-18 antibody only reduced most cell subsets of splenocytes, but not of peripheral blood (Figure 4). Furthermore, the total number of leukocytes in periodontium were elevated ~10-fold in 2-week LPS injection + *P. gingivalis* infection mice and 4-week *P. gingivalis* infection mice compared with controls, corresponding with the increase in all specific cell subsets (Figure 4). Anti-IL-18 antibody administration decreased the number of total leukocytes by 6.5-fold. The number of all cell subsets infiltrated was also reduced in these mice such that their numbers were similar to or even lower than those detected in controls (Figure 4).

**Figure 5:**
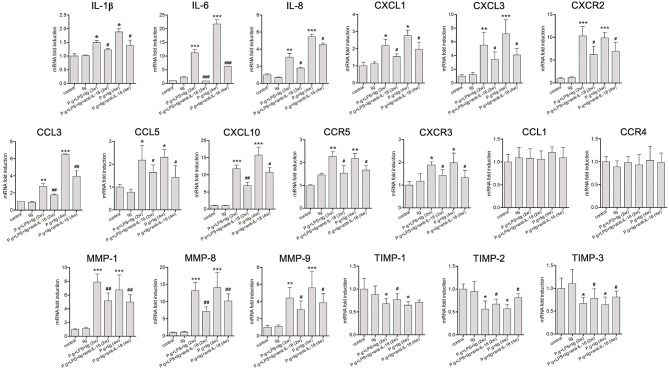
IL-18 depletion modulates cytokine and chemokine expression in the periodontium. Expression of proinflammatory cytokines, chemokines, chemokines matrix metalloproteinase (MMP), and tissue inhibitor of metalloproteinase (TIMP) in the periodontium from untreated control mice (*n* = 3), silk ligature mice (*n* = 3), mice given LPS injection and *P. gingivalis* infection for 2 weeks (*n* = 6), mice with anti-IL-18 antibody administration (*n* = 4), mice given *P. gingivalis* infection for 4 weeks (*n* = 5), and mice with anti-IL-18 antibody administration (*n* = 4). RNA expression was measured by quantitative reverse-transcription polymerase chain reaction, and the results are displayed as fold differences relative to the control groups, normalized to β-actin. Data are expressed as mean ± standard deviation. Group differences were assessed by SNK-*q* tests. *P < 0.05, **P < 0.01, ***P < 0.001 compared with controls. ^#^*P* < 0.05, ^##^*P* < 0.01, ^###^*P* < 0.001 compared with mice without anti-IL-18 antibody administration. lig, ligation; *P.g., P. gingivalis*.

A number of factors, including cytokines, chemokines, MMP, and tissue inhibitors of metalloproteinase (TIMP), are known to be important for the infiltration of cells into the periodontium in periodontitis (23). Therefore, we examined the influence of IL-18 on periodontal cytokine/chemokine expression (Figure 5). Consistent with the changes in inflammatory cell recruitment (Figure 4), LPS injection and *P. gingivalis* infection induced the elevation of proinflammatory cytokines (IL-1β, IL-6, and IL-8), neutrophil chemoattractants (CXCL3, CXCL1) and their receptor (CXCR2), and Th1-type chemokines (CCL3, CCL5, CXCL10) and their receptors (CC5R, CXCR3). Expression of these cytokines and chemokines was reduced in the presence of anti-IL-18 antibody (Figure 5). However, neither *P. gingivalis* infection nor anti-IL-18 antibody administration affected the expression of Th2-type chemokine CCL1 and its receptor CCR4 (Figure 5). Moreover, the quantitative analysis of MMPs mRNA expression in periodontium from *P. gingivalis* infected mice showed a significant increase when compared with controls. Conversely, the expression of TIMPs was found to be generally decreased in *P. gingivalis* infected mice (Figure 5). Administration of anti-IL-18 antibody down-regulated MMPs but slightly up-regulated TIMPs expression in *P. gingivalis* infected mice (Figure 5).

## Discussion

To the best of our knowledge, this is the first study which assessed the newly identified lymphocyte subset NKB cells in periodontitis patients, and functionally analyzed the NKB cells secreting cytokine IL-18 in the immune response to *P. gingivalis*-induced periodontitis *in vitro* and *in vivo*. NKB cells, which could be detected in both peripheral blood and periodontium of humans and mice, were increased in periodontitis patients and *P. gingivalis*-infected periodontitis murine model. This might indicate bacterial infection-induced elevation of tissue-resident NKB cells and recruitment of antigen non-specific immune cells into the periodontium. NKB cells played a vital role in the eradication of microbial infection through IL-12 and IL-18 production (5). However, we only found IL-18, but not IL-12, was elevated in peripheral blood and GCFs of periodontitis patients. IL-18 could be produced by both monocytes/macrophage and NKB cells. We showed that IL-18 was mainly secreted by NKB cells in periodontitis patients, suggesting the important role of NKB cells secreting IL-18 in periodontitis. Recombinant IL-18 stimulation *in vitro* did not affect the bioactivity of periodontal ligament cells but promoted proinflammatory cytokine production by these cells. Furthermore, IL-18 neutralization *in vivo* not only suppressed *P. gingivalis*-induced alveolar bone loss, but also blocked the infiltration of antigen-non-specific immune cells as well as production of cytokines, chemokines, and MMPs in the periodontium. The present data revealed potential proinflammatory activity of NKB cells secreting IL-18 in periodontitis.

NKB cells appeared to be one of the first responders to microbial infections, however, whether NKB cells represented a stable and unique lymphocyte subset or in fact are just the subpopulation of conventional B cells has been heavily debated. NKB cells could activate NK cells and innate lymphoid cells upon stimulation, leading to the modulation of an innate and adaptive immune response for controlling bacterial and viral infections (5). However, Kerdiles et al. (24) questioned whether murine NKB cells displayed the phenotypic and functional characteristics of conventional B cells, and thus might not actually be a separate subset of B cells. In rebuttal, Wang et al. (6) suggested mRNA expression of NK1.1 and NKp46 within NKB cells in mice. The findings in the present study supported the existence of NKB cells as an independent cell population. Phenotypically, CD3^−^CD19^+^ cells expressing NK cell-specific markers (NKp46 or NK1.1) were found in the blood and periodontium of both humans and mice, which was consistent with the previous report which showed that CD3^−^CD20^+^NKG2A^+^ phenotyping was identified as NKB cells in rhesus macaques (25). Moreover, NKB cells were systemically found, but only expand in the gastrointestinal tract during simian immunodeficiency virus infected rhesus macaques. However, human immunodeficiency virus infection did not induce the expansion of peripheral NKB cells in humans (25). A more recent study by Ge et al. (26) suggested that alcohol increased NKB cells' proportion and serum IL-18 expression in an experimental alcoholic liver injury murine model. One of the findings in the present study was the striking expansion of NKB cells in circulation and periodontium during periodontitis. Although NKB cells were putatively considered as the rapid responders to infectious pathogens, we noticed that peripheral and periodontal-infiltrating NKB cells were also expanded in both acute and maintenance phases of periodontitis patients, as well as in chronic *P. gingivalis* infected mice. This might not only indicate long-term expansion of NKB cells in response to bacterial infections, but also suggests the association between intraoral infection and systemic immune disorders. Moreover, elevated periodontal-infiltrating NKB cells positively correlated with the degree of periodontal supporting tissue destruction, revealing the possible contribution of NKB cells to periodontitis, although we could not detect periodontal-infiltrating NKB cells in patients after effective periodontal therapy. Collectively, we speculated that NKB cells were a distinct subpopulation to conventional B cells sharing several properties overlapping with NK cells (5, 25), although the function of expanded NKB cells in periodontitis remained unclear.

IL-18 and IL-12 served as two major cytokines secreted by NKB cells upon microbial challenge. IL-18 was constitutively expressed in NKB cells and robustly increased in response to infection. However, IL-12 was not constitutively expressed in NKB cells and only moderately elevated during microbial infection (5). We found that only IL-18 was elevated in the serum and GCFs of periodontitis patients, and effective periodontal therapy dampened IL-18 production in GCFs, indicating the signature role of periodontium-resident IL-18 in the pathogenesis of periodontitis. Previous studies have revealed that IL-18 could directly inhibit viral replication *in vitro* (27) and protect mice from bacterial and viral infection (5, 28). More importantly, IL-18 not only played a critical role in the induction of IFN-γ from Th1 cells, NK cells, and innate lymphoid cells, but was also involved in Th2, IL-17-producing γδT cells, and macrophage activation (29, 30). Thus, IL-18 is an important regulator in different types of immune cells. Our current results indicated that the proinflammatory function of IL-18 had an effect on the intra-periodontium inflammatory response that followed the *P. gingivalis* infection. *In vitro* study showed that IL-18 stimulation did not affect the cell cycle, apoptosis, or proliferation of purified periodontal ligament cells from healthy individuals, but did substantially promote proinflammatory cytokines' production by periodontal ligament cells, suggesting that IL-18 did not directly regulate cell growth within the periodontium but modulated the immune response in periodontitis. Furthermore, neutralization of IL-18 not only inhibited alveolar bone resorption, but also significantly reduced the cytokine and chemokine expression and the subsequent recruitment of inflammatory cells into the periodontium. IL-18 may therefore directly or indirectly contribute to periodontal disease by promoting the migration of inflammatory cells (31), which are known to induce periodontitis, into the periodontium. The infiltrating of inflammatory cells required specific cellular and protein mediators including neutrophils (32, 33), chemokines (34, 35), and MMPs (36–38). IL-18 has also been shown to promote neutrophil recruitment (39) and induce cytokine/chemokine (40) and MMPs expression (41), which may account for the proinflammatory effect in the *P. gingivalis*-induced periodontitis murine model.

There were several animal models for recapitulation of periodontitis. The present periodontitis murine model was mainly induced by *P. gingivalis* infection and LPS stimulation. Although a silk suture was used, it only slightly applied to the cervical region. This was not similar with traditional ligature-induced periodontitis. *P. gingivalis* infection exacerbated the destruction of alveolar bone induced by circumference ligature in rats (42). Moreover, Suh et al. (43, 44) showed that the combination of silk suture ligature and *P. gingivalis*-originated LPS injection mediated periodontitis-like lesions after 9 weeks. Thus, ligature-induced periodontitis required tight suture ligature around the tooth, which was important for mechanically-induced periodontal inflammation, dental plaque accumulation, and oral mucosal ulceration (43). As shown in the current study, slight application of silk suture did not induce artificial wounding of the periodontium, and only confirmed the adhesion of *P. gingivalis*. Thus, bacterial infection and secretory toxins mainly contributed to the present periodontitis murine model, which accurately simulated the natural process of human periodontal diseases. However, studies using murine models might invoke potential bias due to operations (45). Further experiments using various methods and more research into statistical correction might be useful to deal with the bias (45).

There were several limitations in the present study. Firstly, signaling through IL-18/IL-18R induces the activation of two major pathways, including myeloid differentiation factor 88 (MyD88)/IL-1 receptor-associated kinase (IRAK)/TNF receptor associated factor 6 (TRAF6) pathway and signal transducer and activator of transcription (STAT)/mitogen-activated protein kinase (MAPK) pathway (46, 47). These two pathways promote the expression of nuclear factor-κB, leading to the proinflammatory response. However, we did not characterize the pathways that are potentially involved and link IL-18 and MMPs/TIMPs in *P. gingivalis*-induced periodontitis due to the limited sample size. Secondly, IL-18 could be secreted by both immune cells and parenchymal cells. Administration of anti-mouse IL-18 antibody neutralized total IL-18. Further studies will focus on the specific IL-18 depletion within NKB cells during periodontitis.

In summary, NKB cells-secreting IL-18 may potentiate the intra-periodontal infiltration of antigen non-specific inflammatory cells that can increase subsequent alveolar bone resorption. The tissue-residential distribution and the proinflammatory property may be significant for making NKB cells and IL-18 unique targets in the development of immunotherapeutics for periodontitis.

## Data Availability Statement

The raw data supporting the conclusions of this article will be made available by the authors, without undue reservation.

## Ethics Statement

The studies involving human participants were reviewed and approved by Ethics Committee of Hospital of Stomatology, Sun Yat-sen University (Guangzhou, Guangdong Province, China) [Approval No. ERC-(2016)-39]. The patients/participants provided their written informed consent to participate in this study. The animal study was reviewed and approved by Institutional Animal Care and Use Committee of Sun Yat-sen University (Guangzhou, Guangdong Province, China) (Approval No. SYSU-IACUC-2019-000320).

## Author Contributions

JT contributed to conception and design, analysis and interpretation, and drafted the manuscript. YZ, WK, and DL contributed to conception and design, acquisition, analysis and interpretation, and drafted the manuscript. YL, YF, XL, and CH contributed to acquisition, analysis and interpretation, and drafted the manuscript. G-BH contributed to analysis and interpretation and critically revised the manuscript. J-QL and X-FY contributed to conception and design, analysis and interpretation, and critically revised the manuscript. YX and SX contributed to analysis and interpretation and drafted the manuscript. All authors gave final approval and agree to be accountable for all aspects of the work.

## Conflict of Interest

The authors declare that the research was conducted in the absence of any commercial or financial relationships that could be construed as a potential conflict of interest.
