# Supplementary material for: Natural Killer-Like B Cells Secreting Interleukin-18 Induces a Proinflammatory Response in Periodontitis
Source: Front Immunol. 2021 Feb 18;12:641562. doi: 10.3389/fimmu.2021.641562 (PMC7930384; doi:10.3389/fimmu.2021.641562)
Supplement: Supplementary file 1 [file Data_Sheet_1.PDF]

Table S1. Clinical data of enrolled periodontitis patients

	Acute phase (*n*=12)	Maintenance phase (*n*=26)	*P* value
Age (years)	47.92 ± 8.73	48.46 ± 10.70	0.879
Sex (Male/Female)	9/3	18/8	0.572
Clinical attachment level (mm)	4.84 ± 0.63	4.18 ± 0.65	0.005
Probing depth (mm)	5.74 ± 0.93	4.78 ± 1.11	0.013
Bleeding on probing (%)	100%	88.46%	0.134

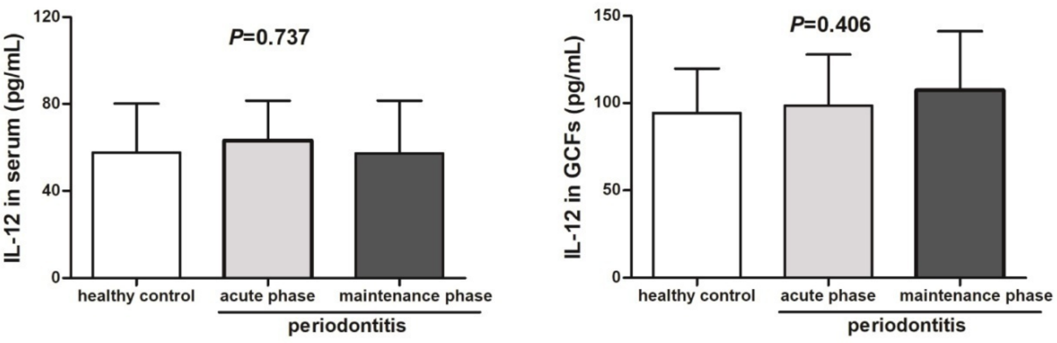

Figure S1. Comparison of IL-12 concentration in serum and GCFs among groups (One-way ANOVA).

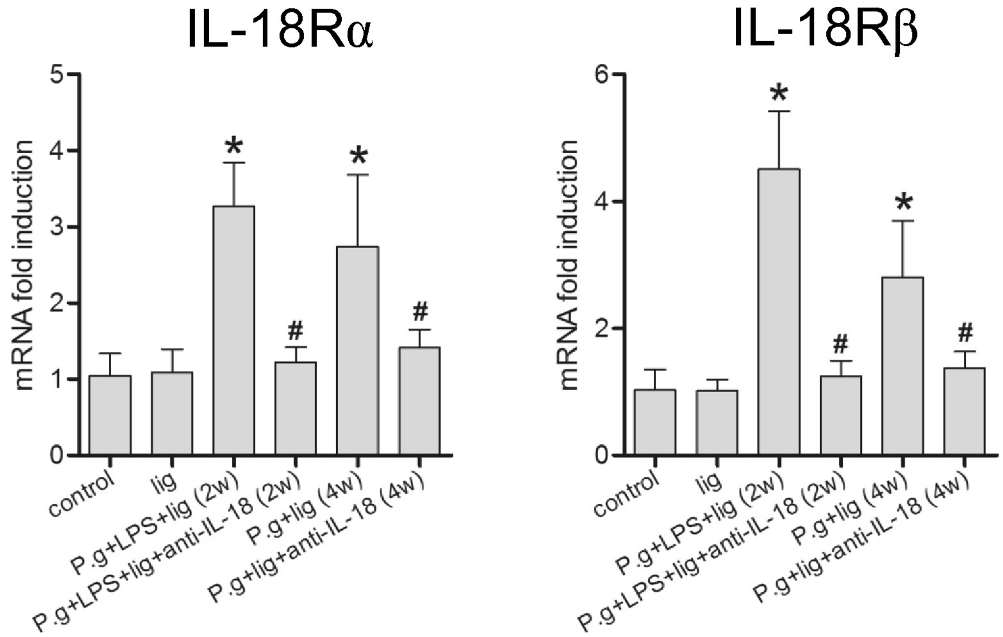

Figure S2. IL-18 depletion modulates IL-18 receptor (IL-18R) expression in the periodontium. Expression of IL-18Rα and IL-18Rβ in the periodontium from untreated control mice (*n*=3), silk ligature mice (*n*=3), given LPS injection and *P. gingivalis* infection for 2 weeks (*n*=6), and along with anti-IL-18 antibody administration (*n*=4), given *P. gingivalis* infection for 4 weeks (*n*=5), and along with anti-IL-18 antibody administration (*n*=4). RNA expression was measured by quantitative reverse-transcription polymerase chain reaction, and the results are displayed as fold differences relative to the control groups, normalized to β-actin. Data are expressed as mean ± standard deviation. Group differences were assessed by SNK-*q* tests. * $P<0.05$, ** $P<0.01$, *** $P<0.001$ compared with controls. $^{\#}$ $P<0.05$, $^{\#\#}$ $P<0.01$, $^{\#\#\#}$ $P<0.001$ compared with mice without anti-IL-18 antibody administration. lig: ligation, *P.g.*: *P. gingivalis*.

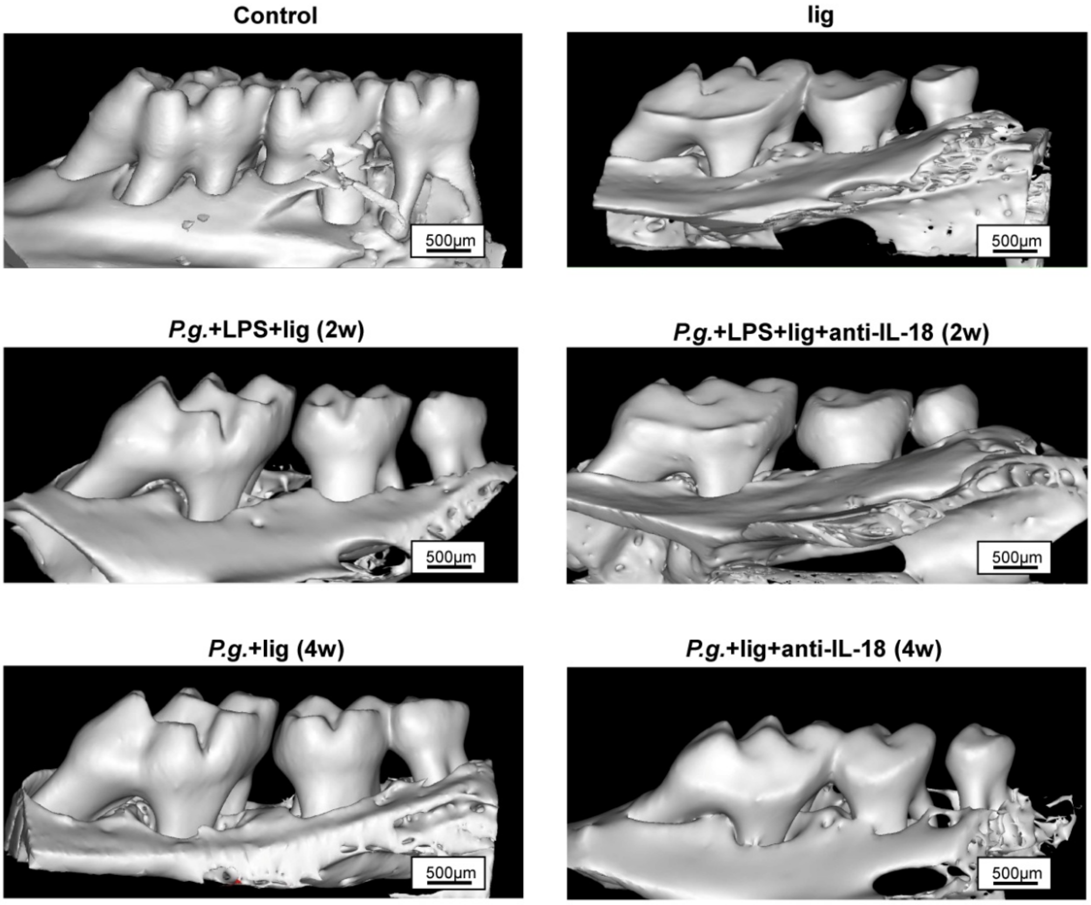

Figure S3. Representative micro-CT three-dimensional images viewed from palatal side of maxillae in untreated control mice (*upper left panel*, *n*=3), silk ligature mice (*upper right panel*, *n*=3), given LPS injection and *P. gingivalis* infection for 2 weeks (*middle left panel*, *n*=5), given *P. gingivalis* infection for 4 weeks (*lower left panel*, *n*=4), and with anti-IL-18 antibody administration (*lower left panel*, *n*=3; *lower right panel*, *n*=3).
